# Large-scale silicon photonics switches for AI/ML interconnections based on a 300-mm CMOS pilot line

**DOI:** 10.1515/nanoph-2025-0475

**Published:** 2025-11-24

**Authors:** Keijiro Suzuki, Ryotaro Konoike, Siim Heinsalu, Shu Namiki, Hitoshi Kawashima, Kazuhiro Ikeda

**Affiliations:** 13508 National Institute of Advanced Industrial Science and Technology (AIST), Tsukuba, Japan

**Keywords:** optical switches, integrated photonic circuits, AI, machine learning, silicon photonics

## Abstract

Silicon photonics switches are emerging as a key technology for realizing energy-efficient networks, spanning from intra data center to wafer-scale interconnections. This review focuses on recent developments and prospects of silicon photonics switches operating in the O-band, which is widely used in computing networks designed for artificial intelligence and machine learning applications. We first review our recent works on O-band silicon photonics switches fabricated by 300-mm silicon photonics technology. Specifically, we have expanded the port count of our O-band switches from 8 × 8 to 32 × 32 implemented with double Mach–Zehnder switch elements for a broad operating bandwidth. This switch achieved a 70-nm bandwidth for a crosstalk of less than −20 dB, and an average on-chip loss of 11.8 dB. Next, we discuss switch topologies optimized for wafer-scale interconnection. Conventional switch topologies typically have their input and output ports at opposite ends of the switch matrix, respectively, which poses challenges of long propagation distances and many waveguide intersections for off-chip planar waveguide routing to connect xPUs on substrate. To address this, we propose a topology where input and output ports are placed adjacently. An O-band 8 × 8 switch based on this topology was fabricated and experimentally demonstrated. Finally, we discuss the prospects and challenges of silicon photonic switches. Key issues include insertion loss, switching speed, crosstalk and operating bandwidth, and polarization dependence. These aspects are examined with reference to reports from other research groups, highlighting both current limitations and potential directions for further improvement.

## 1 Introduction

In recent years, the amount of data processing and traffic inside data centers has grown rapidly due to the increasing use of artificial intelligence (AI) and machine learning (ML), resulting in significant increase of power consumption [1]. Large-scale AI model training and inference using NVIDIA’s GPUs, as well as Google’s TPUs (Tensor Processing Units), have greatly increased the need for communication between servers and racks. Because of this trend, many researchers are concerned that networks based only on electrical switches may not be able to keep up in terms of power efficiency and scalability. As a result, optical switches are now being seriously considered as a practical solution. For example, Google has already introduced optical switches in its large-scale data centers as spine switches in leaf-spine network architecture to improve energy efficiency and cost [2], [3]. They have also been used in the TPU v4 supercomputer [4]. One important feature of optical switches is that their power usage depends only on the number of ports, and not on how fast the signals are. This makes them suitable for low-power, high-bandwidth communication, which is essential for future data center networks. As another trend, processing large-scale AI models requires multiple processor chiplets mounted on larger package substrates. TSMC announced that their system on wafer technology is expected to be ready for large-scale production by 2027 [5]. For such a wafer-scale substrate with longer interconnect distances, reconfigurable (optical switching) wafer-scale optical interposers with a higher bandwidth density and energy efficiency have been proposed and developed [6], [7].

Optical switches can be divided into two main types: free-space optics (FSO) based and photonic integrated circuit (PIC) based. FSO-based switches offer excellent optical performance (loss, crosstalk, etc.), while they are slow in switching (in milliseconds), physically large, and expensive mainly due to assembling cost. On the other hand, PIC-based switches may not exhibit optical performance as same as those of FSO-based, but they are much faster (less than several microseconds), very compact, and low-cost. Some studies suggest that switching times below millisecond can bring real benefits for managing traffic in data centers [8], [9]. Also, when considering wafer-scale optical substrates, only PIC-based switches are practical due to speed, space, and manufacturing constraints. Several device platforms have been used for PIC-based optical switches, including silicon [10], [11], [12], [13], silicon with micro-electro-mechanical systems (MEMS) [14], [15], [16], and Indium Phosphide (InP) [17], [18], [19], with many reports available for each. Among these, we believe silicon photonics stand out because of their excellent process uniformity, high integration density, mass productivity, and applicability of advanced packaging technologies for electronics.

This paper summarizes recent progress in silicon photonics switches for AI/ML applications. We begin with a brief overview of the silicon photonics switches we have developed, followed by a detailed description of our recently reported 32 × 32 switch operating in the O-band [20]. For intra data center interconnects, operation in the O-band is particularly desirable, which offers low dispersion in standard single-mode fibers and eliminates the need for costly dispersion compensation techniques such as digital signal processing. Next, we discuss the optimization of switch topology for wafer-scale interconnection [21]. We point out a critical issue in applying conventional optical switches to wafer-scale interconnection and propose a new switch topology to address the challenge. Finally, we discuss future directions and challenges in the development of silicon photonics switches, including recent works from other research groups.

## 2 O-band silicon photonics switches fabricated with a 300-mm CMOS pilot line

In this section, we summarize the silicon photonic switches we have developed using our 300-mm silicon photonics technology. Our facility is equipped with an ArF immersion lithography system for 45-nm CMOS prototyping and has been used for a series of silicon photonics processes on 300-mm silicon-on-insulator (SOI) wafers [22]. The high-resolution ArF immersion lithography and ultra-uniform 300-mm wafers for advanced volume production are of particular importance for large-scale PICs with ultrahigh uniformity and low loss. Using the pilot line, we fabricated a 32 × 32 switch that operates in the O-band, which was described in detail in our previous work [20]. We summarize its key aspects here and then introduce a new switch topology designed for future wafer-scale interconnects [21]. Since wafer-scale interconnects do not use optical fibers, a new topology is needed where input and output ports are placed next to each other.

### 2.1 32 × 32 silicon photonics switch

We have continuously developed silicon photonic switches with the path independent insertion loss (PILOSS) topology [23]. While various switch topologies [24] exist, such as crossbar, Benes, double-layer network (DLN), switch-and-select, and so on, PILOSS offers several advantages. It is strictly nonblocking, meaning that establishing a new path does not interfere with existing connections. In principle, the insertion loss remains constant regardless of the selected path, enabling simple control from a network perspective. Moreover, the simple two-dimensional matrix layout of the switch allows high-density electrode placement on the chip. It also facilitates individual calibration of element switches by monitoring the output ports. These implementation and control benefits are among the key reasons we have adopted this topology.

Initially, we reported an 8 × 8 switch [25], [26] operating in the C-band and subsequently expanded the port count to 32 × 32 [27]. To reduce insertion loss, we optimized the device design and adopted high-Δ silica PLC connectors, achieving an average fiber-to-fiber insertion loss of 10.8 dB. We also addressed broadband operation across the entire C-band and polarization independence, successfully implementing both features simultaneously in an 8 × 8 switch with a compact FPGA control board [28]. Although the above results were obtained in the C-band designs, we have also reported an 8 × 8 switch operating over a wide bandwidth in the O-band [29]. Most recently, we have reported 32 × 32 switch operating in the O-band with broadband characteristics [20]. We describe its fabrication, implementation, and optical performance evaluation in the following subsections.

#### 2.1.1 Fabrication, packaging, and control

**Figure 1:**
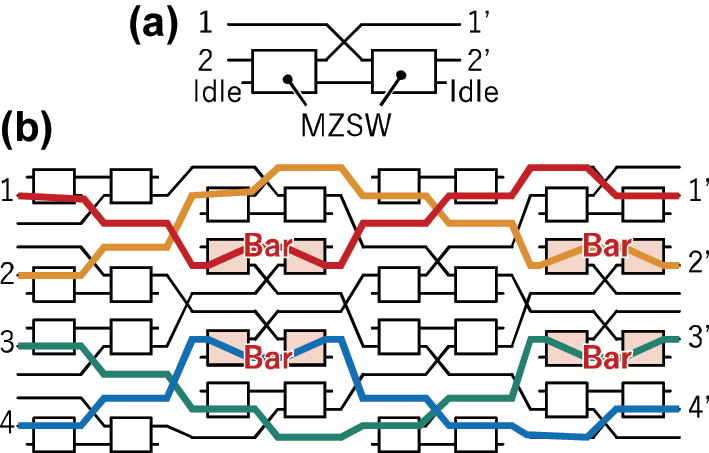
Path-independent insertion loss topology based on double-MZ switch element. (a) Configuration of double Mach-Zehnder (MZ) switch element. (b) Example for a 4 × 4 port configuration. The red, orange, green, and blue lines indicate path 1-–1′, 2–2′, 3–3′, and 4–4′, respectively.

The 32 × 32 switch adopts the PILOSS topology, utilizing double Mach–Zehnder (MZ) switch elements [28], [30] to improve crosstalk and its operating bandwidth. The configuration of the double-MZ switch element, and a 4 × 4 example of a PILOSS switch matrix employing this switch element, is depicted in Figure 1(a) and (b), where each double-MZ switch is designed to operate in the normally cross state.

In Figure 1(a), when the double-MZ switch is in the cross state, both individual MZ switches operate in the bar state. In this configuration, input port 1 is connected to output port 2′, and input port 2 is connected to output port 1′. Since port 2′ is connected to the output port of the switch matrix, leakage light from port 2 to port 2′ contributes to the overall crosstalk. This leakage is attenuated twice by the MZ switches in the bar state within the double MZ switch structure, which effectively suppresses crosstalk.

On the other hand, when the double-MZ switch is in the bar state, both MZ switches are in the cross state. Light from port 2 is directed to port 2′. Light from port 1 is routed to the idle port of the rear MZ switch. However, this route is unused. No light is inputted into port 1 when the double MZ switch is in the bar state. This is because, in the PILOSS configuration, the paths do not intersect at the bar state switch.

When constructing the PILOSS topology using the double-MZ switch, the switches are arranged with alternating orientations in both the row and column directions, as illustrated in Figure 1(b). In this configuration, the overall crosstalk of the switch matrix is primarily determined by the intersections. We have developed and employed an optimized adiabatic intersection structure that achieves excellent performance [29].

**Figure 2:**
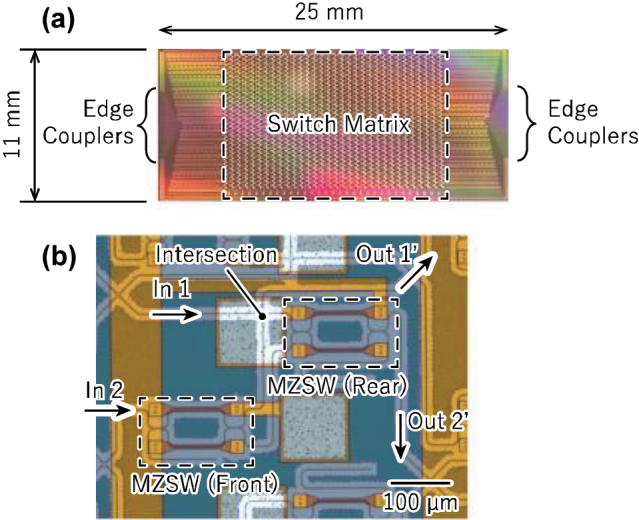
Microscope image of fabricated switch. (a) Chip. (b) Enlarged image of fabricated double Mach-Zehnder switch element.

We designed the 32 × 32 switch for the TE mode and fabricated it by using our 300-mm silicon-photonics pilot-line described before. Figure 2(a) shows the fabricated switch chip with a chip size of 11 mm × 25 mm. The switch matrix consists of 1,024 double-MZ switch elements (i.e., 2,048 MZ switches) and 1,985 intersections. Edge couplers for input and output are placed at both ends of the chip. Figure 2(b) shows a micrograph of double-MZ switch element. Directional couplers were employed as the 3 dB couplers in each MZ switch, whose insertion loss is much lower than that of multimode interference couplers. The thermo-optic (TO) phase shifters based on TiN heaters have a time constant of approximately 30 μs, which can be reduced to a few microseconds using the turbo-pulse technique [31], [32].

For electrical packaging, we employed a flip-chip bonding technique. The chip contains 2,114 electrode pads for controlling and grounding the 2,048 MZ switches, which need to be fanned out and connected to the control board. The switch chip was flip-chip bonded to a ceramic interposer with 0.5-mm pitched land-grid-array (LGA). After attaching optical fiber arrays to the switch chip, the interposer was inserted into a 2,114-pin socket mounted on the control printed circuit board (PCB). The control PCB incorporates five field programmable gate arrays (FPGAs), enabling individual control of all MZ switches on the chip. These FPGAs generate electrical pulses at a repetition rate of 1 MHz, and the power delivered to each MZ switch heater is adjusted by tuning the duty cycle of the pulses. The total power consumption when all input ports are connected to their designated output ports was estimated to be 27.3 W. Of this, 26.0 W was used for calibrating the initial phase errors of the MZ switches, and 1.3 W was required to switching. In the fabricated 32 × 32 switch, only one arm of each MZ switch was electrically wired due to a limitation in the number of interposer electrodes. As a result, roughly half of the MZ switches required phase shifts exceeding 2π for phase trimming, leading to increased power consumption. If both arms of the MZ switches could be controlled, the total power consumption is expected to be reduced to approximately 10 W. Note that our fabricated MZ switch elements have nearly equal arm lengths, making them insensitive to ambient temperature fluctuations that almost equally change the temperature of both the arms. Therefore, no temperature control or cooling is required.

In the fabricated 32 × 32 switch, 16 out of the 2,048 MZ switches failed to operate as described in the next subsection. This issue is attributed to the disconnections at the flip-chip bonding and the contact failures between the interposer and the socket. Therefore, improvements in the parts and processes are necessary. Controlling both arms of each MZ switch by doubling the number of electrodes to ensure redundancy is also under consideration. Although this would require approximately 4,000 electrodes, recent semiconductor packaging technologies have exhibited the capability to handle up to 4,938 electrodes [33], making this approach a feasible option. Other potential solutions include reducing the number of control electrodes via time-division control [34] or monolithically integrating the control electronics.

For optical packaging, two 38-port optical fiber arrays were bonded to the edge coupler arrays at both edges of the chip. High numerical aperture fibers with a mode field diameter (MFD) of 4.0 μm at a wavelength of 1.31 µm were used in the fiber array. The fiber array was actively aligned to minimize coupling loss at both ends of the chip and subsequently fixed in place using UV-curable adhesive.

Even when using 300-mm silicon photonics technology, initial phase errors between the two arms of each MZ switch are present. Therefore, it is necessary to calibrate these phase errors so that the double-MZ switch elements operate in the cross state (i.e., connections 1–2′ and 2–1′ in Figure 1(a)). The details of this calibration procedure are summarized in the references [35], [36]. In essence, electric power values to set each MZ switch to the bar and cross states are individually identified by varying the input electric power to its thermal phase shifter while monitoring the output power at the corresponding port along the connected path. To avoid interference from light leaking through nonintended paths, it is essential to calibrate the MZ switches in a right order. Given that the 32 × 32 switch contains 2,048 MZ switches, manual calibration is impractical. To address this, we developed and executed a semi-automated calibration program, excluding input port fiber reconnections and polarization adjustments. The total calibration time was approximately 20 h, primarily limited by the communication speed between the control PC, FPGA, and optical power meters. As an example, using a commercially available FPGA together with a monolithically integrated photodetector, 50 measurements can be completed in just 0.4 s [37]. If we build a similar system and apply the recently reported calibration algorithm [35], which requires an average of 7.2 measurements per MZ switch, the calibration of 2,048 switches can be completed in approximately 120 s (calculated as ∼2,048 × 7.2/50 × 0.4).

#### 2.1.2 Optical performance

The optical loss of the fabricated switch was measured for all paths. Light from a cw tunable laser set to a wavelength of 1,322 nm, the center wavelength determined by evaluating test MZ switches fabricated on the same wafer, was coupled into the switch via an optical fiber. The switch was controlled to activate only one target path during each measurement, and all other switches were powered off. The 32 output ports were monitored using four 8-channel optical power meters, and the fiber-to-fiber insertion loss was recorded. The on-chip loss was then calculated by subtracting the coupling loss of 5.5 dB/facet, which was obtained from a coupling loss test waveguide fabricated on the same chip.

**Figure 3:**
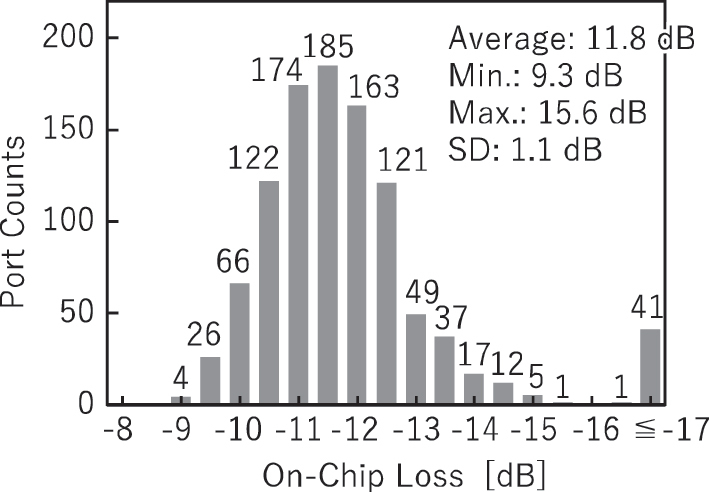
On-chip loss distribution of all paths at a wavelength of 1,322 nm.

Figure 3 shows the distribution of the measured on-chip loss for all paths. Paths that include nonfunctional switches exhibit higher losses. Excluding these paths, the average, minimum, maximum, and standard deviation of the on-chip loss were 11.8 dB, 9.3 dB, 15.6 dB, and 1.1 dB, respectively. Due to the inherent characteristics of the PILOSS topology, all optical paths should exhibit uniform loss. Random variations in the core width may occur across the large die area of 25 mm × 11 mm due to fabrication imperfections. However, we confirmed that the loss variation caused by the random variations is small. In the evaluation of separate chips with many test devices and edge couplers fabricated uniformly, we obtained a small loss variation across the chip (at least smaller than the variation in coupling loss for fiber arrays). Therefore, the observed variation in insertion loss is primarily attributed to fluctuations in coupling efficiency between the optical fibers and the chip, which may be caused by misalignment or bent of the silicon chip, the fiber array, or both, and by variation in the fiber positions within the fiber array. Such variations will be mitigated by using other connection methods such as a silica PLC connector [10], [38]. Since the silica PLC connector can integrate pitch conversion to a narrower pitch, the impact of such variation can be reduced.

Based on the evaluation results of individual test devices fabricated on a separate chip, the detailed loss breakdown for the path with the minimum on-chip loss is summarized in Table 1. As shown in Table 1, propagation loss accounts for a substantial portion of the total loss. Therefore, further reduction of propagation loss is essential for minimizing overall insertion loss.

**Table 1:** Breakdown of minimum on-chip loss.

Components	Loss
Routing waveguide from input edge coupler to switch matrix	1.1 dB (=0.67 cm × 1.67 dB/cm)
Waveguides connecting MZ switches	2.9 dB (1.72 cm × 1.67 dB/cm)
Mach–Zehnder switches	2.8 dB (33 × 0.085 dB)
Intersections	1.3 dB (62 × 0.021 dB)
Routing waveguide from switch matrix to output edge coupler	1.2 dB (=0.72 cm × 1.67 dB/cm)

**Figure 4:**
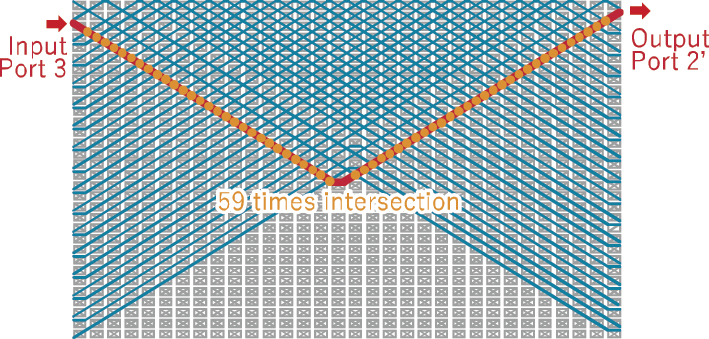
One of the worst crosstalk paths. Path connection setting: input 1 – output 32′, 2–30′, 3–2′, 4–28′, 5–27′, …, 28–4′, 29–3′, 30–29′, 31–1′, and 32–31′. Path 3–2′ has 59 crossings with other paths.

Next, we evaluated the crosstalk of the fabricated switch. Since the number of switch configurations is 32 factorials, it is infeasible to perform measurements for all possible states. Therefore, we evaluated the worst-case crosstalk scenario, in which the selected optical path intersects with the largest number of other paths. One such configuration is illustrated in Figure 4, where the path from port 3 to port 2′ intersects with the other 31 paths a total of 59 times. To evaluate crosstalk, we first measured the transmitted power through path 3–2′. Subsequently, we configured the switch to have both path 3–2′ and another target path. Light was then injected into the input port of the target path, and the leaked power to output port 2′ was measured. This procedure was repeated for the other 31 paths while varying the wavelength.

**Figure 5:**
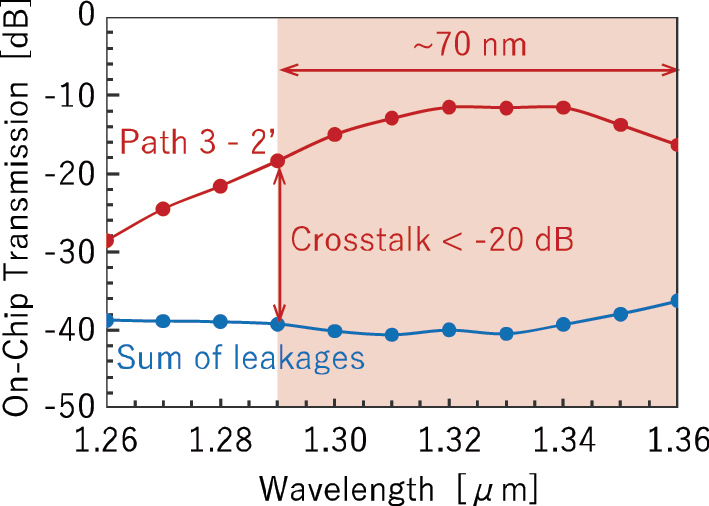
On-chip transmission spectrum of path 3–2′ and sum of leakages spectrum from other paths to port 2′.

Figure 5 shows the transmission spectrum of the measured path from port 3 to port 2′, along with the wavelength dependence of the total leaked optical power from the other 31 paths to output port 2′. Here, crosstalk is defined as the ratio between these two values. A crosstalk level below −20 dB was achieved for wavelengths of longer than 1.29 µm, with a corresponding bandwidth exceeding 70 nm. Although leakage light from other paths is suppressed over a broad wavelength range, it is necessary to broaden the transmission bandwidth of the path for broadband signal formats such as CWDM4 [39].

Table 2 summarizes the above results in comparison with the key characteristics of previously reported silicon photonic switches operated in the O-band. Various optical switches have been demonstrated using different switching mechanisms and topologies [12], [16], [20], [40]. Among them, our 32 × 32 switch represents one of the largest port counts reported to date. The insertion loss is also relatively low among devices fabricated purely with silicon photonics. Regarding crosstalk, other groups measured the extinction ratio, which is defined as the ratio between the transmitted power in a given path and the highest leaked power to other output ports. In contrast, our measured crosstalk represents performance in more practical operation fully loaded by optical signals. We define it as the ratio between the transmitted power in a given path and the total leaked power from all other paths into that path. Our evaluation method is more stringent, and our switch showed lower crosstalk over a wider spectral range compared to those reported by other groups.

**Table 2:** Comparison of strictly nonblocking silicon photonics switches operating in the O-band.

Switch technology	Port count	On-chip loss	Crosstalk and its bandwidth	Switching power	Switching time	Footprint	Ref.
Double layer with EO switch	8 × 8	7.5–10.5 dB	Not provided (30 nm for −20 dB ER^a^)	Few mW	<10 ns	12 mm × 7 mm	[12]
Crosspoint with waveguide MEMS	32 × 32	8.2 dB	Not provided	Not provided (40 V)	Sub µs	4 mm × 4 mm (estimated)	[16]
Switch-and-select with microring	4 × 4	4.5 dB	Not provided (0.35 nm for −28.3 dB ER^a^)	Not provided	Not provided	Not provided	[40]
PILOSS with TO MZ switch (our work)	32 × 32	11.8 dB	>70 nm for −20 dB	18 mW	Few µs	11 mm × 25 mm	[20]

^a^ER, extinction ratio that defined as the ratio between the transmitted power along the desired optical path and the maximum power leaked into any other port.

### 2.2 Switch topology optimization for wafer-scale interconnection

In this subsection, we discuss a switch topology optimized for wafer-scale interconnection. We first identify a challenge in conventional switch topologies for wafer-scale interconnection and propose a modified version of the PILOSS topology as a solution. We then present experimental results demonstrating the topology using an 8 × 8 switch.

#### 2.2.1 Input and output ports adjacent topology

**Figure 6:**
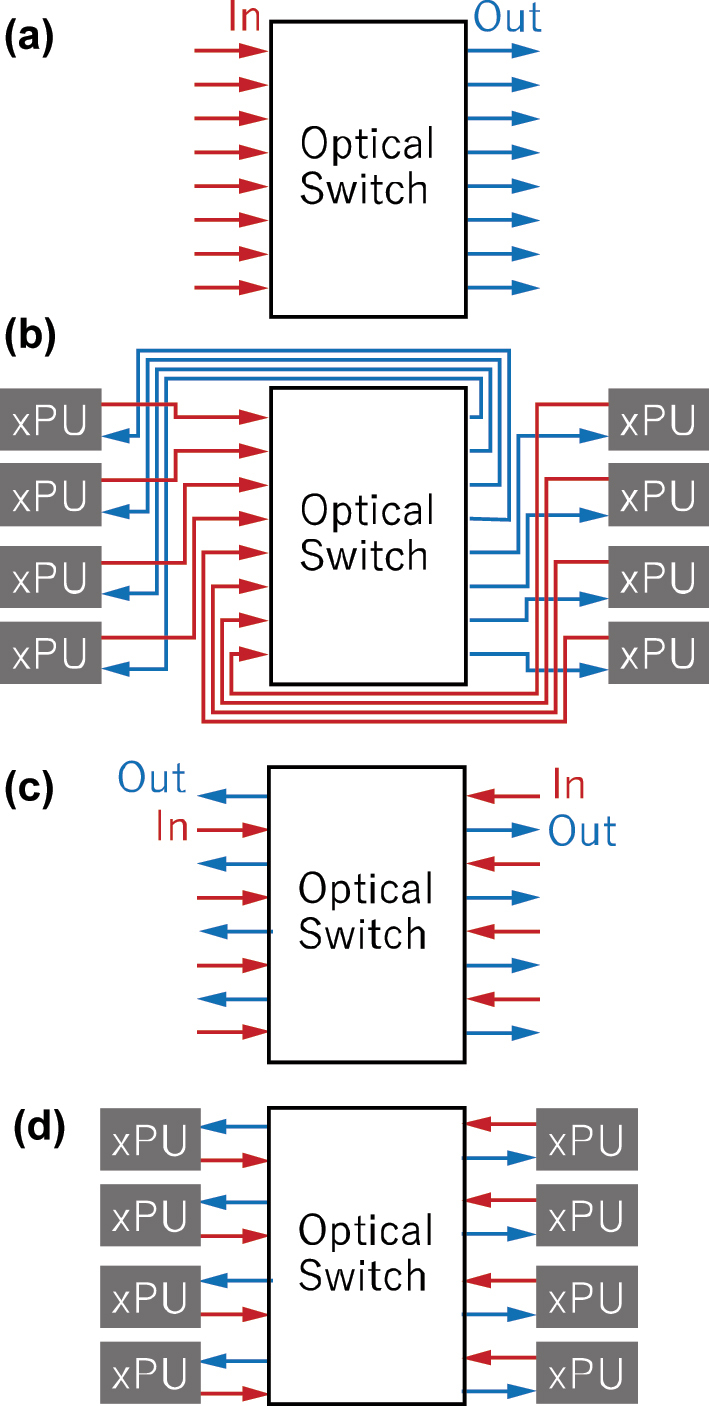
Comparison of xPU connections in conventional optical switches and in switches with adjacent input/output ports. (a) Input and output port arrangement of conventional switch topologies. (b) Example of interconnection between xPUs implemented on the same plane using a conventional switch topology with standard input/output port placement. (c) Input/output port arrangement in the proposed switch topology, where input and output ports are placed adjacent to each other. (d) Wiring configuration between xPUs when using the proposed switch topology.

Figure 6(a) illustrates the input/output port arrangement in conventional switch topologies, where input and output port arrays are placed at the opposite ends. This configuration causes no problems for systems in which optical fibers are used to connect switches and nodes, such as intra/inter data center, and telecom networks. However, this arrangement can pose a challenge in wafer-scale optical interconnects, where many xPUs are interconnected with planar optical waveguides. Figure 6(b) presents an example connection in which eight xPU chiplets are connected through an 8 × 8 optical switch with the conventional input/output port arrangement. An extra length of waveguide and a number of waveguide intersections are required to connect the xPU chiplets to the switch, which lead to increased insertion loss and crosstalk as the port-count increases. To address this issue, switch topologies in which input and output ports are placed adjacent to each other can be used, as illustrated in Figure 6(c) [21]. With this port arrangement, xPU chaplets can be interconnected using simplified wiring, as shown in Figure 6(d). This configuration eliminates the need for long optical waveguide routing and many waveguide intersections, thereby mitigating insertion loss and crosstalk. Following the above observations, we propose a new switch topology, i.e., input and output ports adjacent PILOSS.

**Figure 7:**
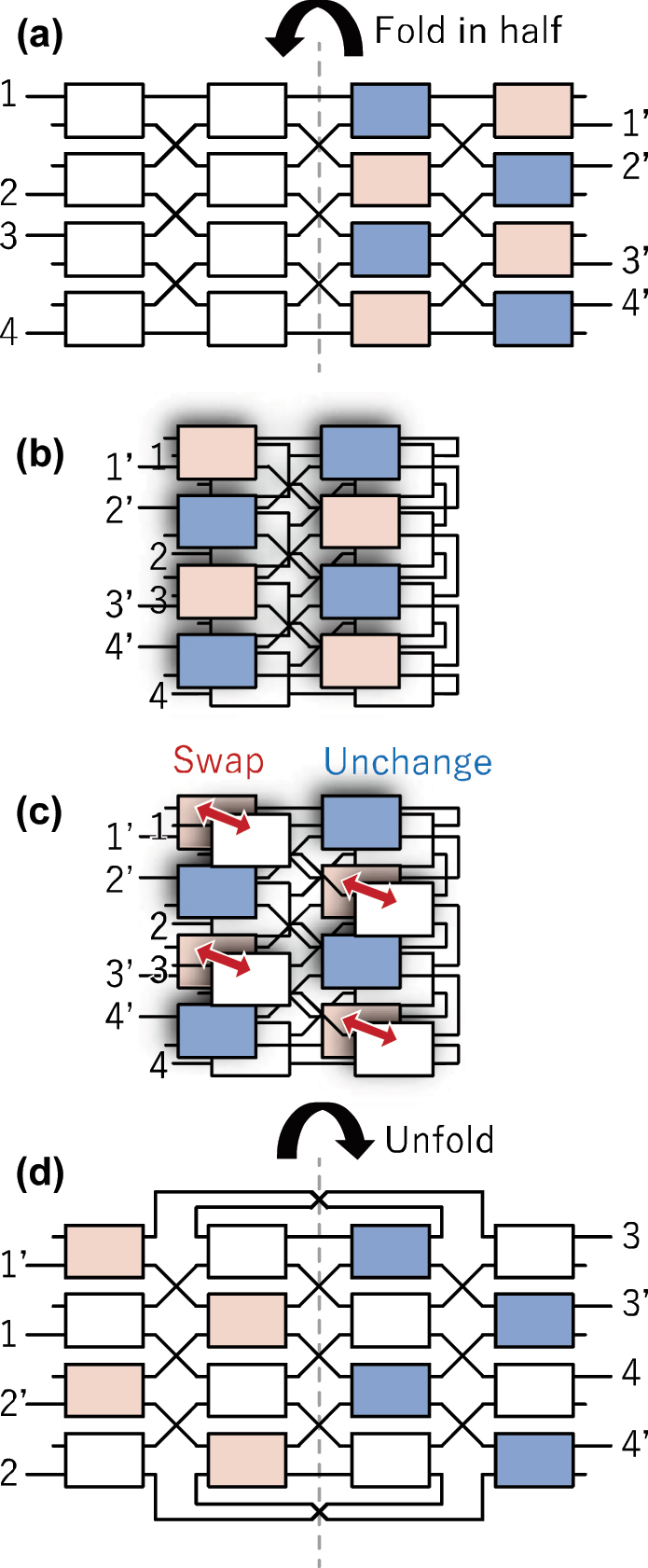
Transformation procedure from the conventional path-independent insertion loss (PILOSS) topology to the adjacent input/output port topology. (a) Configuration of the 4 × 4 PILOSS topology. The light red and blue regions in the rear half are used as visual markers. (b) The 4 × 4 switch shown in (a) folded in half. (c) In the folded section, the light red region is swapped with the switch located directly below, while the light blue region remains unchanged. (d) The switch is unfolded after the swapping operation described in (c).

For simplicity, consider a 4 × 4 PILOSS topology shown in Figure 7(a), where the rear half of the element switches are colored for reference. First, the switch is folded in half along the center line while preserving the connectivity between element switches, as illustrated in Figure 7(b). Next, among the colored switches, those marked in light red are brought to the back of the nonmarked switches located directly beneath them, while the light blue ones remain unchanged, as shown in Figure 7(c). Finally, the switch is unfolded back to its original layout, and the input/output ports are renumbered, resulting in the configuration shown in Figure 7(d). The new switch topology has their input and output ports adjacent to each other. Table 3 summarizes the structural differences between the conventional and proposed PILOSS topologies. The number of element switches on a single path, as well as the number of switches operating in the bar state, remains unchanged. Also, the number of waveguide crossings per path is almost the same (*N* − 1 and *N* − 2). Thus, the adjacent port configuration introduces no disadvantages. If any drawback is to be noted, it would be a need for longer routing waveguides on the outer side of the switch. Nevertheless, since these waveguides can mostly be implemented as straight segments, they can be designed with widened low loss structures and are, therefore, not expected to have a significant impact on overall performance.

**Table 3:** Comparison of proposed topology with conventional one.

	Conventional PILOSS	Input/output port adjacent PILOSS
Switches on a path	*N*	*N*
Bar-state switches on a path	1	1
Intersections on a path	*N* − 1	*N* − 2

#### 2.2.2 Experimental demonstration

**Figure 8:**
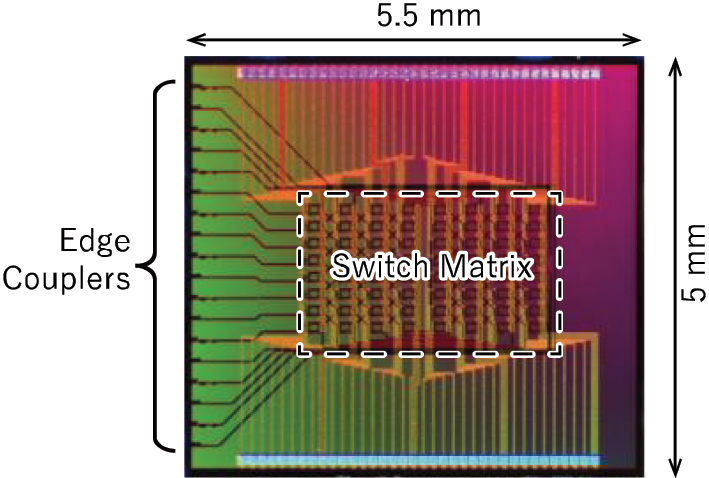
Fabricated chip of input and output ports adjacent 8 × 8 switch.

To experimentally validate the proposed topology with adjacent input/output ports, we fabricated an 8 × 8 switch with the topology. Figure 8 shows an optical microscope image of the fabricated chip. Similar to the 32 × 32 switch described in the previous subsection, the device was designed for the TE-mode and the O-band and fabricated using our 300-mm silicon photonics pilot line. Each element switch is a heater-controlled MZ switch. To reduce the number of electrodes, a standard single MZ switch element was used instead of the double-MZ switch element. The fabricated chip was die-bonded onto a ceramic chip carrier, and wire bonding was used to connect the electrode pads on the chip to those on the carrier. The carrier electrodes were routed through a PCB to a 64-channel digital analog converter (DAC) array. Electrical power supplied to each switch heater was controlled via a PC connected to the DAC array. For optical interfacing, edge couplers were grouped on one side of the chip, and an 18-channel high-NA (MFD: 4.0 μm at a wavelength of 1.31 µm) fiber array was butt-coupled to them. Based on evaluation of test patterns, the coupling loss between the fiber and chip was measured to be 4 dB/facet. Similar to the 32 × 32 switch, the 8 × 8 switch also requires calibration of the initial phase errors between the arms of each MZ switch. While the basic procedure remains the same, the 8 × 8 switch contains only 64 MZ switches, making manual calibration sufficiently feasible.

**Figure 9:**
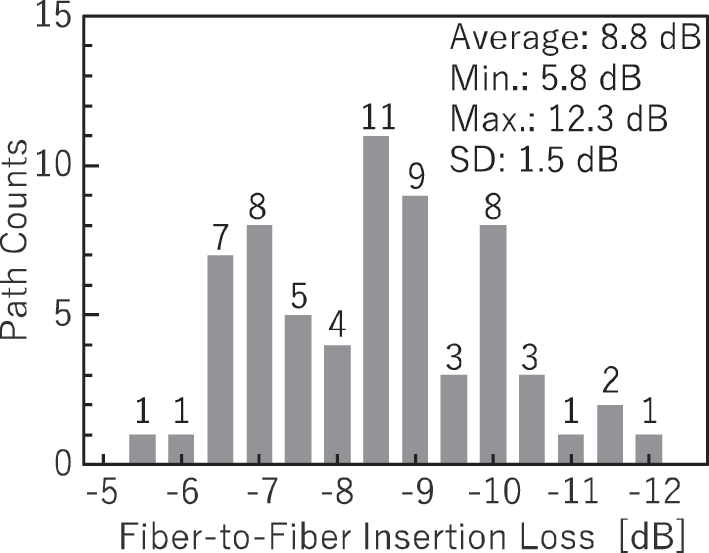
Distribution of fiber-to-fiber insertion loss of all paths.

Using a tunable laser diode and an 8-channel optical power meter, we measured the fiber-to-fiber insertion loss of all paths. The results are shown in Figure 9. The average, minimum, maximum, and standard deviation of the fiber-to-fiber insertion loss were 8.8 dB, 5.8 dB, 12.3 dB, and 1.5 dB, respectively. As with the 32 × 32 switch, this variation is primarily attributed to variation in coupling loss between the optical fibers and the chip. According to the port-by-port loss distribution, the coupling loss was lower near the center of the fiber array and tended to increase toward the edges. This suggests that either the fiber array, the chip, or both may be slightly bent at the center.

**Figure 10:**
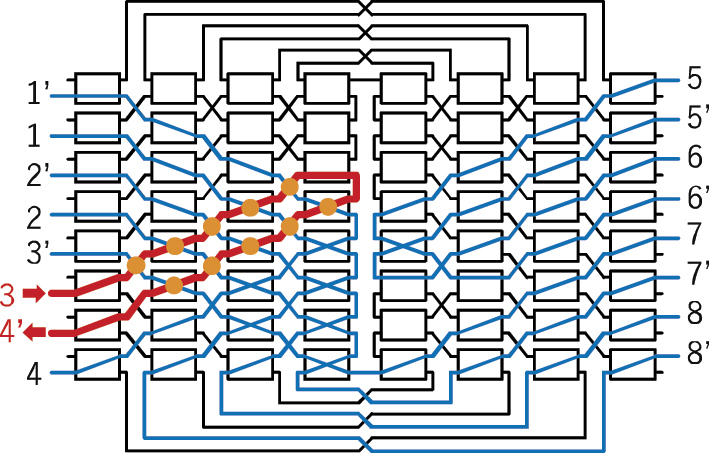
One of the worst crosstalk paths. Path connection setting: input 1 – output 8′, 2–7′, 3–4′, 4–1′, 5–6′, 7–3′, and 8–2′. Path 3–4′ has 10 crossings with other paths.

**Figure 11:**
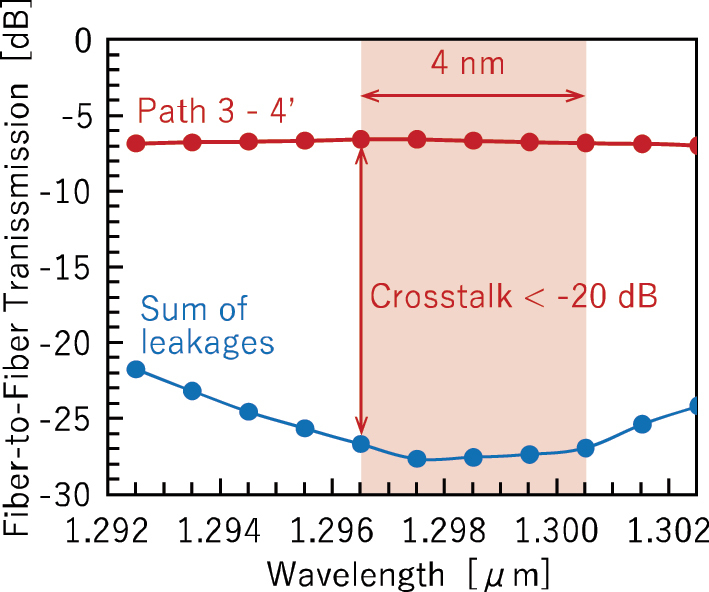
On-chip transmission spectrum of path 3–4′ and sum of leakages spectrum from other paths to port 4′.

Next, we evaluated crosstalk. Figure 10 illustrates one of the worst crosstalk paths within all paths of the proposed 8 × 8 switch. The path from input port 3 to output port 4′ traverses the left half of the switch matrix and folds back, crossing with the other paths a total of ten times. Figure 11 presents the transmission spectrum of path 3–4′, along with the wavelength-dependent sum of leaked light from the other paths that outputs at port 4′. Defining crosstalk as the ratio between these two spectra, the wavelength range in which the crosstalk remains below −20 dB was found to be 4 nm. Due to limitations in the number of available electrodes, single MZ switch elements were employed in this 8 × 8 switch. This resulted in relatively high crosstalk and a narrow operational bandwidth. If double-MZ switch elements were used instead, as demonstrated in our previous 8 × 8 switch [29], it is expected that a crosstalk of −30 dB could be achieved over a bandwidth exceeding 70 nm.

## 3 Outlook and challenges of silicon photonics switches

This section discusses the remaining challenges for practical deployment and potential directions for performance improvement of silicon photonics switches from the perspectives of insertion loss, switching speed, crosstalk, and polarization independence.

### 3.1 Loss

Insertion loss remains one of the most significant challenges toward the practical deployment of silicon photonics switches. Currently, Google reports that the optical switches deployed in its data centers exhibit insertion losses typically less than 2 dB [2], which serves as a benchmark for practical intra data center networks. In contrast, the O-band 32 × 32 switch we demonstrated exhibits an average on-chip loss exceeding 10 dB, indicating a substantial gap from the target performance.

The primary sources of loss are the propagation loss in silicon waveguides and the coupling loss between silicon waveguides and optical fibers. Reducing the propagation loss requires further optimization of the fabrication process, such as minimizing the line edge roughness of the silicon waveguide core. Additionally, widening the waveguide core may help mitigate scattering at the sidewalls, although careful design is necessary to suppress higher-order mode excitation depending on the structure. Direct coupling between silicon waveguides and optical fibers remains challenging due to significant differences in mode field diameter and mode shape, even when spot-size converters are employed. Therefore, the use of high-Δ silica PLCs [38] or similar intermediate structures with such as SiN [41], SiON [42], metamaterials [43], etc. is considered a more practical solution.

Another potential approach to reduce effective loss is to introduce optical gain for loss compensation. Previously, we demonstrated hybrid integration of semiconductor optical amplifiers (SOAs) in a 4 × 4 switch [44], [45]. Lossless operation was confirmed over a wavelength range from 1,510 nm to 1,565 nm, and crosstalk was measured to be below −34 dB across a similar bandwidth. In addition, successful transmission of 8-channel, 32 Gbaud, 16 QAM signals was verified [44]. For large-scale integration, such as 32 × 32 ports, a large-scale SOA array would be required, posing challenges in thermal management and packaging. Successive integration of two gain chips having 4 SOAs was demonstrated for an 8 × 8 multicast switch, toward large-scale integration [46].

**Figure 12:**
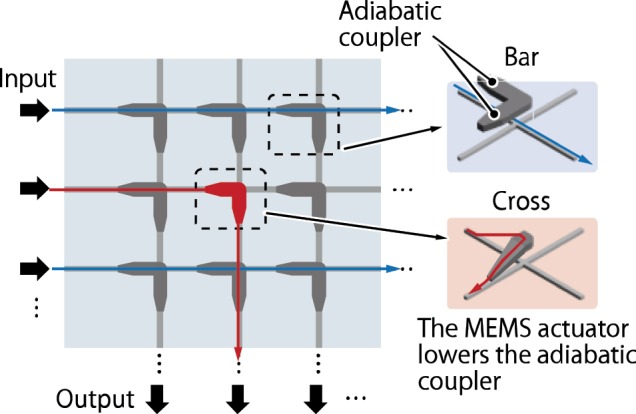
Crossbar switch that employs MEMS actuated element switch. The switch consists of a bus waveguide layer and an upper switching waveguide layer. The MEMS actuator lowers an adiabatic coupler in the upper layer.

As a different approach, the use of MEMS as element switches has also been reported [16]. The structure of the switch is illustrated in Figure 12. The switch topology is a crossbar configuration where silicon waveguides are arranged in a mesh-like bus circuit with orthogonal crossings, and on the upper layer, L-shaped switching elements composed of two adiabatic waveguide couplers are implemented on the cross points. By actuating the adiabatic waveguide couplers made of polycrystalline silicon via MEMS and bringing them closer to the underlying bus waveguides, the optical path can be switched from the horizontal to the vertical bus waveguide, thereby enabling signal routing. Using this method, switches with up to 240 × 240 ports operating in the C-band was fabricated, and a maximum insertion loss of 9.8 dB was measured [15]. The switching time is reported to be in the submicrosecond range. However, packaging these MEMS switches remains technically challenging. Due to the presence of movable MEMS components, existing mature packaging technologies developed for electronic circuits cannot be directly applied, and new development efforts are required. A 32 × 32 port scale implementation has been reported while the operation of all switch elements has not yet been fully verified [16].

### 3.2 Switching speed

Most of our switches utilize the TO effect induced by TiN heaters. The typical time constant of the TO phase shifter is approximately 30 μs [27]. By optimizing the shape of electrical pulse (a technique we refer to as “turbo pulse” technique), we achieved switching within a few microseconds [31]. When heating one of the heaters, the switching speed can be improved by applying a short pulse of high power. In contrast, since active cooling of the heater is difficult, we accelerate the switching during the cooling phase by heating the heater in the opposite arm, which induces a phase shift in the reverse direction. Switching at even shorter timescales, in the nanosecond range, can be realized using electro-optic (EO) phase shifter using the carrier plasma effect in PIN structures. We fabricated an 8 × 8 PILOSS switch using the EO phase-shifters [47]. In our design, each arm of the MZ switch integrates a TO phase shifter for initial phase error compensation and an EO phase shifter for switching. If the switch matrix were constructed using EO phase shifters alone, EO elements would also be used for initial phase error compensation, leading to additional loss and crosstalk due to carrier absorption and amplitude unbalance between two arms of MZ switches, respectively. Since we adopted the PILOSS topology, each optical path has only one EO phase shifter in the ON state (i.e., with carrier injection and associated loss), which is advantageous in terms of uniform and minimal insertion loss. Regarding crosstalk, leakage from an MZ switch in the ON state is directed to the idle ports of the PILOSS switch matrix and, therefore, does not affect the overall crosstalk performance. Using this switch architecture, we demonstrated switching faster than 8 ns. Although these results were obtained in the C-band, the switch can be designed for the O-band.

**Figure 13:**
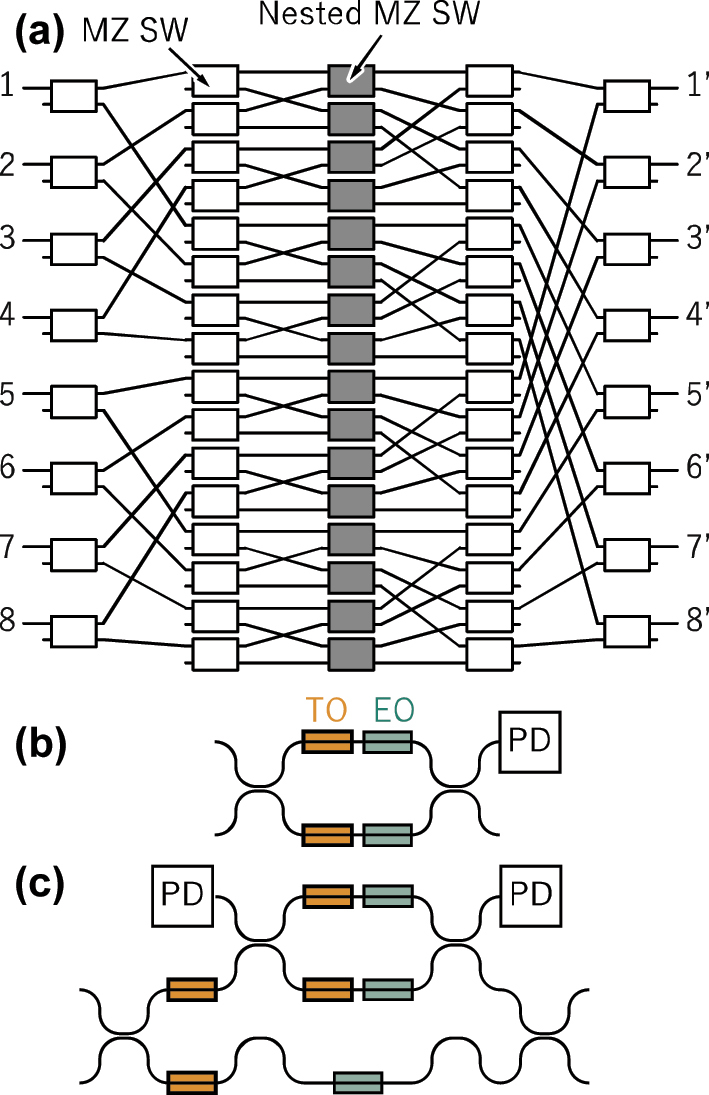
Double-layer network using a nested MZ switch. (a) Network topology. (b) Mach-Zehnder switch. TO: thermooptic. EO: electrooptic. PD: photodetector. (c) Nested Mach-Zehnder switch.

An 8 × 8 fast switch operating in the O-band was reported by a research group in IBM [12]. The switch topology is based on DLN, as illustrated in Figure 13(a). The switching elements consist of conventional MZ switches (Figure 13(b)) and nested MZ switches located at the central region (Figure 13(c)). Each arm of the MZ switches incorporates a heater for compensating for initial phase errors and a PIN structure for switching. The nested MZ configuration is employed to mitigate crosstalk by balancing optical loss in both arms, which is particularly important due to the inherent loss in the PIN phase shifters. The reconfiguration time, including the control electronics, was less than 10 ns.

### 3.3 Crosstalk and operating bandwidth

In the 32 × 32 switch in Section 2, a crosstalk of −20 dB was achieved over a bandwidth exceeding 70 nm. It should be noted that many of the crosstalk values reported by other research groups [13], [48], [49], [50], [51] are based on a different definition: the ratio between the output power of a target path and the maximum leaked power observed at any unintended output port. This definition does not account for the cumulative leakage from multiple paths, and therefore, our definition imposes a more stringent condition. For high-speed intensity-modulation/direct-detection signal demodulation, a crosstalk of −20 dB is not satisfactory, and further reduction is desirable. As shown in Figure 5, one of the contributing factors to crosstalk degradation is the wavelength dependence of the insertion loss at the target port. This dependence originates from the directional couplers used in the cascaded MZ switches. Replacing them with low loss broadband 3 dB couplers could potentially mitigate this issue and improve overall performance.

From the perspective of individual switch element crosstalk, switches based on MEMS technology offer excellent performance. Since the waveguide physically moves, the leakage light from the switch element is extremely small. However, even when using the MEMS switches, the overall crosstalk in the switch matrix is still dominated by the intersection regions. Therefore, the situation is essentially the same as in the PILOSS switch matrix that employs double MZ switch elements where primal crosstalk occurs at the intersections.

### 3.4 Polarization independence

Silicon waveguides inherently exhibit strong polarization dependence. This becomes problematic when transmitting polarization-multiplexed signals, making polarization insensitivity essential. Furthermore, when connected to optical fibers, the input light polarization state becomes random, which also requires polarization-insensitive operation.

There are two approaches to achieving polarization independence: structural polarization insensitivity and polarization diversity. The former requires nanometer-scale uniformity in waveguide geometry due to the high refractive index contrast between silicon and SiO_2_ cladding, making it extremely challenging to implement. As an alternative approach, thick (few micrometers) waveguides [52] or rib waveguides [53] fabricated on thick SOI wafers have been considered. However, this method has not yet achieved sufficient polarization insensitivity, and the device size becomes larger compared to the standard 220-nm-thick SOI platform.

Consequently, the polarization diversity scheme is considered more practical. Using the polarization diversity scheme, we fabricated and demonstrated 8 × 8 switch [54], exhibited a polarization dependent loss of <0.4 dB and a differential group delay of <1.8 ps over 70 nm wavelength range. This method, however, has the drawback of doubling the circuit complexity, as it requires separate switching paths for two orthogonal polarization components. To mitigate this issue, we proposed a nonduplicated polarization diversity topology [55], [56]. In this topology, idle ports, which are normally unused in the PILOSS topology, are utilized. One of the two orthogonal polarizations propagates through the switch matrix in the standard direction, as in conventional PILOSS operation, while the other polarization propagates through the same switch matrix from the opposite direction. Since the PILOSS topology has inherently two synchronous switch circuits, both polarization components can be accommodated at the same time in a single switch matrix, and polarization diversity can be achieved.

Another option is the integration of on-chip polarization controllers [57], which enables real-time compensation of the input polarization state, can be one solution. However, this option can be used only for single polarization signals. Moreover, some challenges, including achieving stable polarization control across the entire O-band, the inability to handle wavelength-dependent polarization variations, and continuously and infinitely tracking the polarization state, remain.

## 4 Conclusions

In this paper, we have summarized recent progress in silicon photonics switches for AI/ML applications. We first provided a brief overview of the silicon photonics switches we have developed, followed by a detailed description of our recently reported 32 × 32 switch operating in the O-band. We then proposed a new switch topology optimized for wafer-scale interconnects, in which input and output ports are placed adjacent to each other, and presented its experimental demonstration. Finally, we discussed future directions and challenges in the development of silicon photonics switches, including recent activities by other research groups.

Unlike electrical switches, optical switches are used for circuit switching and, therefore, cannot simply replace electrical switches for packet switching. To effectively utilize optical switches, it is necessary to integrate them properly into the overall system and develop control technologies on the system side. Google was the first to successfully implement optical switches with such an approach but only for the spine switches. A new project has just begun to openly discuss these challenges at the Open Compute Project [58]. In the future, system-level development will advance, especially for applications in AI and machine learning. It is important to continue developing optical switches while closely following these discussions and technological trends.
